# Transgenic Overexpression of Active Calcineurin in β-Cells Results in Decreased β-Cell Mass and Hyperglycemia

**DOI:** 10.1371/journal.pone.0011969

**Published:** 2010-08-03

**Authors:** Ernesto Bernal-Mizrachi, Corentin Cras-Méneur, Bo Ra Ye, James D. Johnson, M. Alan Permutt

**Affiliations:** 1 Division of Metabolism, Endocrinology, Diabetes, The Brehm Center for Type 1 Diabetes, University of Michigan, Ann Arbor, Michigan, United States of America; 2 Department of Cellular and Physiological Sciences, Department of Surgery, University of British Columbia, Vancouver, Canada; 3 Division of Endocrinology, Metabolism and Lipid Research, Washington University School of Medicine, St. Louis, Missouri, United States of America; University of Bremen, Germany

## Abstract

**Background:**

Glucose modulates β-cell mass and function through an initial depolarization and Ca^2+^ influx, which then triggers a number of growth regulating signaling pathways. One of the most important downstream effectors in Ca^2+^ signaling is the calcium/Calmodulin activated serine threonine phosphatase, calcineurin. Recent evidence suggests that calcineurin/NFAT is essential for β-cell proliferation, and that in its absence loss of β-cells results in diabetes. We hypothesized that in contrast, activation of calcineurin might result in expansion of β-cell mass and resistance to diabetes.

**Methodology/Principal Findings:**

To determine the role of activation of calcineurin signaling in the regulation of pancreatic β-cell mass and proliferation, we created mice that expressed a constitutively active form of calcineurin under the *insulin* gene promoter (*caCn^RIP^*). To our surprise, these mice exhibited glucose intolerance. *In vitro* studies demonstrated that while the second phase of Insulin secretion is enhanced, the overall insulin secretory response was conserved. Islet morphometric studies demonstrated decreased β-cell mass suggesting that this was a major component responsible for altered Insulin secretion and glucose intolerance in *caCn^RIP^* mice. The reduced β-cell mass was accompanied by decreased proliferation and enhanced apoptosis.

**Conclusions:**

Our studies identify calcineurin as an important factor in controlling glucose homeostasis and indicate that chronic depolarization leading to increased calcineurin activity may contribute, along with other genetic and environmental factors, to β-cell dysfunction and diabetes.

## Introduction

The normal response of pancreatic islet β-cells to various conditions associated with Insulin resistance is to increase the mass of Insulin producing cells. Plasma glucose concentration is an important factor in this response and mediates increases in glucose-induced islet β-cell growth and proliferation [1], [2], [3], [4]. In contrast, chronic elevation in plasma glucose, so called glucotoxicity, can have deleterious effects on β-cell function and survival [5], [6], [7], [8], [9], [10], [11], [12], [13]. On the other hand, glucose starvation negatively affects β-cell survival [13], [14], [15]. The explanation for the different responses to glucose levels is unclear but changes in intracellular Ca^2+^ concentrations play an important role. The idea that chronically elevated intracellular Ca^2+^ concentrations due to high glucose can result in deleterious effects on β-cell proliferation, survival and/or function is consistent with the Ca^2+^ set-point hypothesis described in the neuronal literature [16]. This concept states that very low or high intracellular Ca^2+^ levels are incompatible with survival and that between these extremes, Ca^2+^ concentrations have protective and physiological effects on neuronal function.

Increase in intracellular Ca^2+^ by glucose and depolarizing agents activates several intracellular pathways including, Ca^2+^/Calmodulin kinases (CaMK) and extracellular signal-regulated protein kinases (ERK1 and ERK2) and calcineurin among others [17], [18], [19], [20], [21]. Calcineurin is the only serine/threonine protein phosphatase under the direct control of intracellular Ca^2+^ and plays a critical role in coupling Ca^2+^ signals to cellular responses [22]. Therefore, calcineurin is a major candidate to mediate signals activated by glucose-induced depolarization and Ca^2+^ influx. Calcineurin is a heterodimer containing a catalytic/Calmodulin-binding subunit, calcineurin A, tightly bound to a calcineurin phosphatase regulatory Ca^2+^-binding subunit, calcineurin b1 (*Cnb1*) [22]. Calcineurin is an important regulator of multiple biological functions, but very few studies have investigated its role in pancreatic β-cells. Elegant experiments by Heit, *et. al.* demonstrated a role for this signaling pathway in regulation of β-cell growth and function [23]. These studies showed that mice with conditional deletion of *Cnb1* in β-cells developed diabetes as a result of decreased β-cell mass, proliferation and insulin content [23]. This phenotype was associated with decreases in critical genes necessary for β-cell development and function including, *ins1*, *ins2*, *glut2*, *mafA*, *pdx1*, *beta2* and *cyclin D2*. Interestingly, the metabolic phenotype and altered gene expression were restored by conditional expression of active NFATc1 in *cnb1*-deficient β-cells [23]. Nuclear factor of activated T cells (NF-AT) is one of the most recognized calcineurin targets. Moreover, experiments with calcineurin inhibitors FK506 and cyclosporin A (CsA) have provided further insights into the role of calcineurin in metabolism and β-cell function. CsA and FK506 inhibit calcineurin activity by binding to regulatory proteins of the enzyme, Cyclophilin A and FKBP-12 respectively [24]. Administration of CsA and FK506 to rodents [25] or humans [26], [27] induces hyperglycemia and hypoinsulinemia. Complementary *in vitro* experiments *in vitro* using insulinoma cells and human islets have demonstrated that CsA and Fk506 reduce Insulin biosynthesis and secretion [28], [29] [30]. While these studies demonstrated that calcineurin deficiency resulted in β-cell failure and diabetes, it is unclear whether increased glucose-induced Ca^2+^ influx and subsequent calcineurin activation will mimic the hypertrophic effects of chronic depolarization on β-cell function and mass.

The experiments reported herein explored the role of sustained activation of calcineurin activity in regulation of pancreatic β-cell mass and function. To achieve this, we generated transgenic mice overexpressing a constitutively active calcineurin mutant in β-cells under the control of the rat *insulin* promoter. These mice developed hyperglycemia and hypoinsulinemia as a result of decreased β-cell mass and Insulin secretion. The changes in β-cell mass resulted from decreased proliferation and augmented apoptosis. The current work demonstrated that sustained calcineurin hyperactivity negatively impacts β-cell growth and function. These studies imply that calcineurin could mediate some of the glucotoxic effects induced by chronic hyperglycemia in type 2 diabetes.

## Methods

### Generation of transgenic mice

The constitutively active calcineurin used for these experiments lacks the regulatory domain of calcineurin A (CnMut) [31], [32]. The calcineurin mutant was provided by Gerald R. Crabtree (Stanford University School of Medicine) and was generated by introducing a stop codon at nucleotide 1259 as described [31]. This sequence was inserted at the EcoRI site in a RIP-I/β-Globin expression vector. This chimeric gene (*caCn^RIP^*) was excised by enzymatic digestion, purified, and microinjected into fertilized eggs of C57Bl6 × CBA mice according to standard technique. Three transgenic founders (#167, #138 and #139) expressing the *caCn^RIP^* chimeric gene were generated in a C57Bl6 × CBA genetic background. Founders were backcrossed to C57BL6J mice. Experiments were performed on comparable mixed background. Two lines exhibited a similar phenotype. The studies described herein were performed on animals derived from the #138 line. All procedures were approved by the Washington University Animal Studies Committee.

### MIN6 cell culture and adenoviral infection

MIN6 cells were maintained in DMEM (Gibco) as previously described [33]. The cells were transduced either with a control GFP or a constitutively active calcineurin adenovirus overnight at an MOI of 19. The transduced cells were maintained in the media for 48 hours before harvesting.

### Immunoblotting

For western blot analysis, blots of isolated pancreatic islet lysates and MIN6 cells were probed with antibodies against calcineurin A (BD Biosciences), phospho Akt S473 (Cell Signaling) and tubulin (Sigma). Protein obtained from islets (50 g; ∼100 islets) were used for each experiment. Briefly, islet lysates were separated by electrophoresis on polyacrylamide gels and transferred to nitrocellulose or PVDF membranes (Bio-Rad). After blocking overnight, membranes were incubated for 24 hours with primary antibodies at the dilutions recommended by the manufacturer. Immunoblotting experiments were performed at least three times in duplicate.

### Immunostaining, islet morphometry and analysis of proliferation and apoptosis

Pancreata obtained from 12-week-old mice were used for morphometry and immunohistochemistry. Immunostaining for insulin, glucagon, somatostatin and pancreatic polypeptide cells was performed as described [34], [35]. The β-cell mass was calculated by point counting morphometry from 5 insulin stained sections (5 µm) separated by 200 µm using the NIH ImageJ software (v1.43n freely available at http://rsb.info.nih. gov/ij/index.html [36] as described [34], [35]. Pancreata from neonates were obtained during the first 12 hours of life. Proliferation was assessed in insulin and Ki67 (Novocastra, Burlingame, CA) stained sections as previously described [34]. Apoptosis was determined in pancreatic sections using cleaved Caspase 3 (Cell Signaling) and Insulin staining as described [35]. At least 1000 insulin stained cells were counted for each animal.

### Assessment of glucose metabolism and insulin secretion

Fasting blood samples were obtained after overnight fasting from the tail vein. All the metabolic studies were performed in male mice. Glucose was measured on whole blood using AccuChek II glucometer (Roche Diagnostics, Indianapolis). Plasma insulin levels were determined on 5 µl aliquots by using a Rat Insulin ELISA kit (Crystal Chem, Chicago, Illinois). Glucose tolerance tests were performed in 12-hour fasted animals by injecting glucose (2 mg/g) intraperitoneally as described [34].

### Islet isolation and *in vitro* insulin secretion

Islet isolation was accomplished by collagenase digestion and differential centrifugation through Ficoll gradients using a modification of procedures described previously for rat islets [34]. After isolation, islets were hand picked and lysed in lysis buffer (Cell Signaling, Beverly, Massachusetts). Insulin secretion *in vitro* was assessed by static incubation of islets. After overnight culture in RPMI media containing 5 mM glucose, islets of similar size from *caCn^RIP^* mice and wild-type mice were handpicked and pre-cultured for an hour in Krebs-Ringer medium containing 2 mM glucose. Groups of five islets in triplicate were incubated in Krebs-Ringer medium containing either 2 mM glucose, 20 mM glucose, or 30 mM KCl, and incubated at 37°C. After 1-hour incubation, medium was collected and stored at –20°C, after which insulin was measured by RIA. Islet perifusion experiments were carried out as described [37]. Briefly, groups of 80 were suspended in Bio-Gel P2 beads and perifused at 1 mL/min using a temperature-controlled multi-chamber perifusion system (Cellex Biosciences, Minneapolis, MN). Net hormone release responses of perifused cell columns to treatments were quantified by integrating the baseline-subtracted area under the curve during the treatment period. Each time point was subtracted from the prepulse mean, defined as the average of the three time points before the treatment period.

### Statistical analysis

All values are expressed as mean ± SEM. Paired Student's *t* test was used for all comparisons. Differences were considered statistically significant at *p*<0.05.

## Results

### Generation of mice expressing a constitutively active form of calcineurin in β-cells

**Figure 1:**
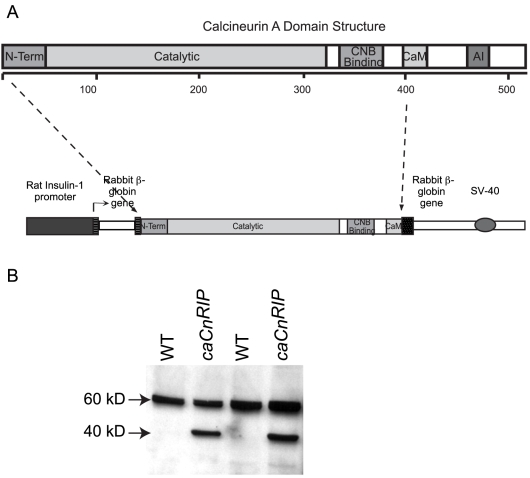
Transgene construct and expression in islet lysates from WT and *caCn^RIP^* mice. **A**. Domain structure of calcineurin A. The mutant form including the first 1259 bp (400 amino acids) was subcloned into a vector containing the rat insulin promoter. **B**. Immunoblotting for calcineurin using islet lysates from WT and *caCn^RIP^* mice. The endogenous calcineurin A band migrates at 60 kD and the caCn mutant at 40 kD. The western is representative of two experiments performed in duplicate.

The constitutively active calcineurin mutant used for these studies lacks the regulatory domain of calcineurin A (caCn) and exhibits Ca^2+^-independent constitutive phosphatase activity *in vitro* and *in vivo* [31], [32], [38]. This was achieved by deleting the carboxy terminal sequence including a fraction of the Calmodulin binding domain and the auto inhibitory domain of calcineurin A as described [31], [32], [38]. This sequence was inserted downstream of the rat *insulin I* promoter sequence (*caCn^RIP^*, Figure 1A). Two lines with a similar expression levels and phenotypes were obtained. The studies described herein were performed on animals derived from one of these lines. Expression of the transgene in islet lysates from WT and transgenic mice demonstrated expression of the mutant protein (40 Kd band) only in *caCn^RIP^* mice (Figure 1B). No alterations in weight were observed in *caCn^RIP^* mice suggesting that there were no major abnormalities in appetite control by expression of the transgene in the hypothalamus (data not shown).

### *caCn^RIP^* mice exhibit hyperglycemia

**Figure 2:**
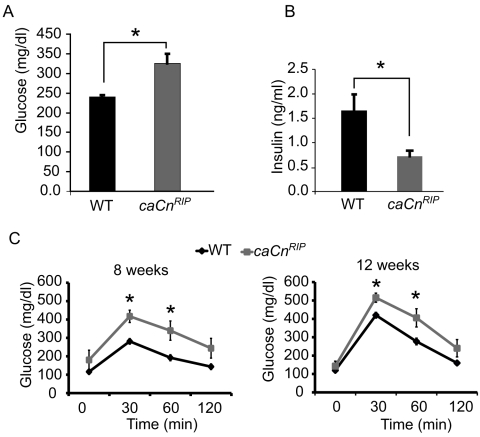
RIP-CnMut mice have higher fed serum blood glucose compared to control littermates. Serum glucose (**A**) and Insulin (**B**) concentrations in non-fasting 2–3 month old *caCn^RIP^* mice (n = 5) and control littermates (n = 8). **C**. Intraperitoneal glucose tolerance tests in 8 and 12-week old *caCn^RIP^* mice (n = 7) and control littermate (n = 4) males as indicated (left and right panels; n = 4). Results are expressed as mean ± SEM. * p<0.05.

To determine the effects of constitutively active calcineurin in islet β-cells on glucose metabolism, we examined random glucose and insulin levels in 8–12 week-old mice. Random glucose levels were higher in *caCn^RIP^* mice (Figure 2A). *caCn^RIP^* mice exhibited concomitant hypoinsulinemia (Figure 2B). Intraperitoneal glucose tolerance testing demonstrated that *caCn^RIP^* mice displayed higher glucose levels after 30 and 60 minutes after glucose injection (Figure 2C). Similar glucose intolerance was observed in 12 week old mice (Figure 2C). The glucose intolerance was also observed in *caCn^RIP^* females (Figure S1).

### Overexpression of constitutively active calcineurin in islets induces the second phase of insulin secretion

**Figure 3:**
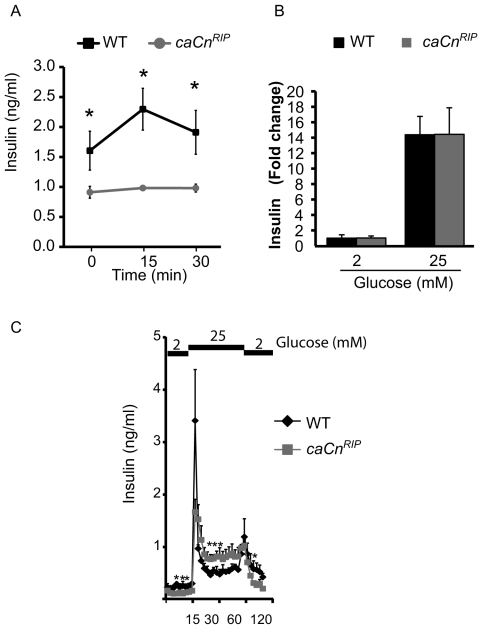
Transgenic mice expressing calcineurin showed altered insulin secretion. **A**. Glucose stimulated insulin secretion *in vivo* assessed by intraperitoneal glucose injection. **B**. Static Insulin secretion in isolated islets from 8–12 week old WT and *caCn^RIP^* mice. **C**. Insulin secretion assessment by islet perifusion experiments with low (2 mM) and high (25 mM) glucose in islets from WT and *caCn^RIP^* mice (*n* = 3). Data expressed as mean ± SEM. *p<0.01.

To begin to elucidate the mechanisms responsible for impaired glucose tolerance in *caCn^RIP^* mice, we assessed insulin secretion *in vivo* and *in vitro*. Insulin levels in *caCn^RIP^* mice were reduced relative to those in WT mice after overnight fasting and did not increase after glucose injection (Figure 3A). Insulin levels were lower in *caCn^RIP^* islets cultured in 2 mM glucose for 60 min (p<0.05, data not shown). Glucose stimulated insulin secretion in isolated islets was similar in *caCn^RIP^* mice (Figure 3B). Since calcineurin signaling has been reported to modulate the different phases of Insulin secretion [39], we performed islet perifusion experiments. Basal Insulin secretion at 2 mM glucose before and after glucose stimulation was decreased in *caCn^RIP^* islets (Figure 3C). No significant difference was observed in the first phase of Insulin secretion (Figure 3C). Interestingly, the second phase of insulin secretion was enhanced in *caCn^RIP^* islets (Figure 3C). However, the area under the curve for glucose stimulated Insulin secretion was comparable (p>0.05, data not shown).

### Pancreas morphometry on WT and *caCn^RIP^* mice

**Figure 4:**
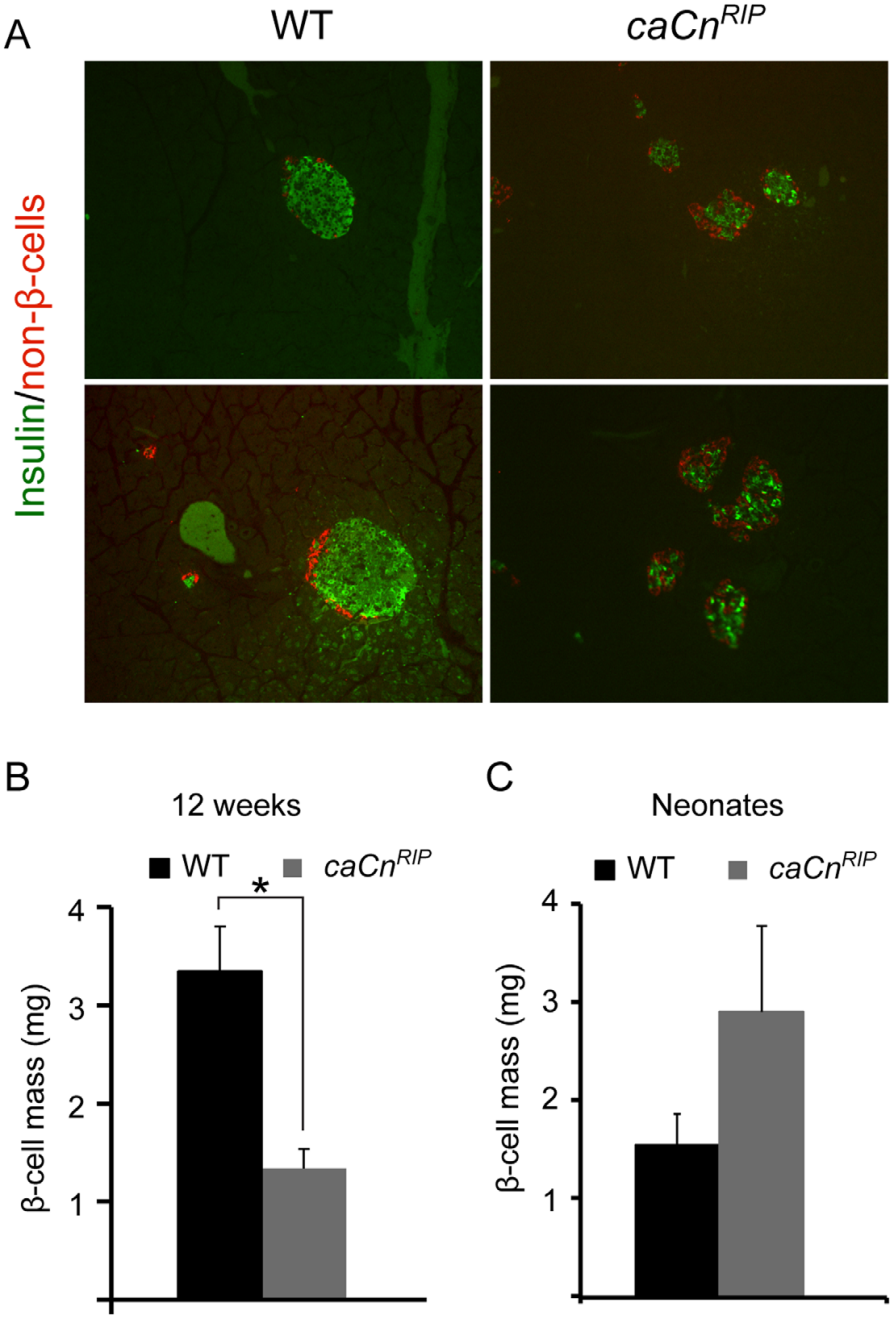
Pancreas morphometry on WT and *caCn^RIP^* mice. **A**. Immunostaining for Insulin and non β-cells in pancreas from WT and *caCn^RIP^* mice. Images presented were obtained at different magnifications (Upper panel 10x and lower panel 40x). Assessment of β-cell mass using point-counting morphometry in WT and *caCn^RIP^* mice at 12 weeks of age (**B**) and neonates (**C**). Results are mean ± SEM (n = 4). * p<0.05.

Morphometric analysis was then performed to determine the cause of altered glucose tolerance and Insulin secretion in *caCn^RIP^* mice. Staining for Insulin and a cocktail of antibodies for non β-cells revealed that islets from *caCn^RIP^* mice showed decreased size and irregular shape (Figure 4A). Analysis of β-cell mass in 12-week-old mice indicated that *caCn^RIP^* mice exhibited more than a 50% reduction in β-cell mass (Figure 4B). To determine whether this reduced mass was a developmental defect or acquired post-natally, the β-cell mass in WT and *caCn^RIP^* neonates was examined and found to be not significantly different (Figure 4C). These studies demonstrate that *caCn^RIP^* mice are born with normal β-cell mass and develop decrease in mass during the first 12 weeks of life.

### Decrease in β-cell mass in *caCn^RIP^* mice results from decreased proliferation and increased apoptosis

**Figure 5:**
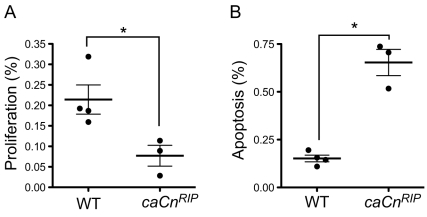
Assessment of proliferation and apoptosis. **A**. Proliferation assessed by BrdU staining in 2–3 month old wild type and mutant mice. **B**. Apoptosis determined by cleaved Caspase 3 staining in wild type and *caCn^RIP^* mice. Data is express as mean + SEM. *p<0.05.

We next examined whether the decrease in β-cell mass in *caCn^RIP^* mice was the result of decreased proliferation or increased apoptosis. Analysis of proliferation performed by Ki67 staining demonstrated that *caCn^RIP^* mice exhibited decreased proliferation (Figure 5A). *caCn^RIP^* mice also displayed a concomitant increase in apoptosis revealed by cleaved-caspase 3 staining (Figure 5B), indicating that calcineurin affects both proliferation and apoptosis.

## Discussion

The current studies extend our knowledge of the role of calcineurin in pancreatic islet β-cells by examining the effects of chronic activation of calcineurin on β-cell mass and function. These experiments demonstrate that long-term activation of calcineurin induces impaired glucose tolerance by alterations in β-cell mass. We also show that activation of calcineurin signaling negatively affects proliferation and survival of β-cells. These morphological alterations resemble in part the phenotype of β-cells exposed to chronic hyperglycemia and suggest that chronic activation of calcineurin could be an important component of the glucotoxic effect of hyperglycemia in type 2 diabetes and possibly also explain the failure of these agents to control diabetes after long-term therapy with this medication.

The expression of the calcineurin mutant in β-cells resulted in major disturbances in plasma glucose levels. A small fraction of the animals developed frank diabetes making it difficult to maintain the line. The abnormalities in glucose were associated with decreased Insulin levels. Transgenic mice exhibited severe impairment in glucose-induced Insulin secretion *in vivo*. The severe defect in Insulin secretion in the context of 50% of normal β-cell mass suggested the possibility that *caCn^RIP^* mice might exhibit some degree of impaired Insulin secretion. However, *in vitro* Insulin secretion in response to glucose was similar in static incubation and perifusion experiments (Figure 3). Islet perifusion experiments showed that islets from *caCn^RIP^* mice exhibited a robust first phase of Insulin secretion implying that the readily releasable pool was not significantly altered. Interestingly, we observed a significant increase in the second phase of insulin secretion suggesting that calcineurin may modulate events associated with insulin granule trafficking [39]. The changes in second phase of Insulin secretion could be explained in part by dephosphorylation of Kinesin Heavy chain (KHC) on β-granules. Phosphorylation of KHC inhibits the binding of granules to microtubules and prevents the transport towards the cell membrane [39], [40]. In contrast, inhibition of calcineurin-mediated KHC dephosphorylation using inhibitors and adenoviruses inhibits second phase of insulin secretion [39]. In summary the discrepancies in insulin secretion *in vitro* and *in vivo* are difficult to reconcile but it is possible that the stress of the isolation procedure and the selection of islets for perifusion are biased to favor the availability and collection of healthier islets and these islets are not completely representative of the integrated response obtained in *in vivo* experiments. It is also probable that activation of calcineurin in neurons could contribute to the regulation of *in vivo* insulin secretion in this model. However, we believe that this is less likely due to lack of evidence of central expression of the promoter used for these experiments.

Decreased β-cell mass was an important component responsible for the hyperglycemic phenotype in *caCn^RIP^* mice. The diminished β-cell mass was caused by reduced proliferation and increased apoptosis. The mechanisms involved in the regulation of β-cell cycle by calcineurin are partially understood. Heit *et al.* showed that transgenic activation of NFATc1 in β-cells induces proliferation by inducing Cyclin D and Cdk4 levels [23]. This suggests that the decreased proliferation observed by activation of calcineurin is mediated in an NFATc1-independent manner. It is important to note that NFAT transcription factors are not the only calcineurin-downstream substrates and other calcineurin-regulated proteins such as Map Kinase Phosphatase 1 (MKP1) [41], Cdk4 [42], [43], PKA, NO synthase and the co-activator TORC could also be involved [44], [45], [46]. To this end, we have demonstrated that glucose and KCl-induced depolarization induces MKP1 expression in a calcineurin-dependent manner (data not shown). Therefore, increased MKP1 can inhibit Mitogen-Activated Protein Kinase (MAPK) activation and subsequent cell cycle progression. In summary, the current findings are consistent with a negative effect of calcineurin on cell cycle progression by activation of downstream signaling targets other than NFATc1. The loss of the transgenic line prevents us from pursuing some of these avenues.

The decreased β-cell mass in *caCn^RIP^* mice could also be explained in part by augmented apoptosis. The role of calcineurin in apoptosis has been extensively examined in neurons and lymphoid tissues, among others [16], [47], [48], [49]. In β-cells, inhibition of calcineurin is protective against apoptosis induced by proinflammatory cytokines and dexamethasone [47], [50], [51]. The mechanisms involved in apoptosis observed in *caCn^RIP^* mice could be multifactorial. As demonstrated in other systems, including β-cells, calcineurin-mediated dephosphorylation and activation of the pro-apoptotic Bcl-2 family protein Bad is a major component of apoptosis induced by elevated Ca^2+^/Calcienurin signaling [49], [50], [51]. Recent experiments showed that calcineurin decreased Akt signaling by dephoshorylation of S473 [52]. However, Akt^S473^ phosphorylation was not altered in MIN6 cells expressing a constitutively active calcineurin suggesting that this mechanism is not likely to play a role (Figure S2). In summary, our studies suggest that the genetic activation of calcineurin signaling reduces β-cell mass by induction of apoptosis. It is unclear at this point if these calcineurin effects are mediated by NFAT.

In summary, the present work shows that chronic activation of calcineurin signaling regulates survival and proliferation of β-cells. These studies together with those obtained in mice with deletion of *Cnb1* in β-cells [23] suggest that calcineurin signaling is a major component of the effects induced by glucose depolarization/Ca^2+^ influx. Similar pattern of responses derived from apoptotic responses to intracellular Ca^2+^ concentration in neurons have led to the development of the Ca^2+^ set-point hypothesis [16]. The results of the present study suggest that persistent activation of calcineurin signaling could be an important component responsible for the responses to chronic depolarization.

## Supporting Information

Figure S1 A Random blood glucose levels in 8-week-old females (n = 4). B. Glucose tolerance in 8-week-old females. Intraperitoneal glucose tolerance tests was performed in 8 and 12-week old caCnRIP mice and littermates females (B) (n = 4).
(0.40 MB EPS)

Figure S2 Western blots on MIN6 cells transduced with a control (GFPAdv) or constitutively active calcineurin (caCnAdv) adenovirus. Immunoblotting for Calcineurin (A) or phospho Akt (473) (B) and tubulin. Expression levels were normalized to tubulin and quantified on the right. Results are mean ± SEM (n = 4). * p<0.05.
(0.71 MB EPS)
